# The effects of the interaction between BMI and dyslipidemia on hypertension in adults

**DOI:** 10.1038/s41598-022-04968-8

**Published:** 2022-01-18

**Authors:** Na Tang, Jian Ma, Rongqin Tao, Zhijun Chen, Yide Yang, Quanyuan He, Yuan Lv, Zelong Lan, Junhua Zhou

**Affiliations:** 1 Key Laboratory of Molecular Epidemiology of Hunan Province, School of Medicine, Hunan Normal University, Changsha, 410013 Hunan China; 2 Tianxin District Center for Disease Control and Prevention, Changsha, 410009 Hunan China

**Keywords:** Diseases, Endocrinology, Risk factors

## Abstract

Body mass index (BMI) and dyslipidemia are indicators of human health and are often associated with high blood pressure. In this study,we explored the relationship between BMI or dyslipidemia and the risk of hypertension and further verified the possible interacting influences of BMI with dyslipidemia on the risk of hypertension. The aim is to explore the possible risk factors of hypertension and to provide scientific basis for the prevention and treatment of hypertension. Eligible subjects were selected from a cross-sectional survey in Changsha City, and we collected relevant data and clinical indicators for each participant. Body mass index (BMI) was calculated as weight (kg)/height^2^ (m^2^), and divided into four categories according to the Chinese standard. Dyslipidemia is defined according to Chinese guideline. Unconditional logistic regression models were used for dichotomous variables to determine the risk or protective factors of dependent variables. Multivariate Logistic model was used to study the influence of BMI and dyslipidemia on hypertension. The following indicators were used to assess the interaction effects: (1) Relative excess risk due to interaction (RERI); (2) Attributable proportion due to interaction(AP); (3) Synergy index (SI). SPSS software was used for statistical analysis. A total of 2740 eligible participants were enrolled in the cross-sectional study, of which 765 subjects (27.9%) were diagnosed with hypertension. Multivariate Logistic model showed that overweight (OR: 1.70, 95%CI: 1.39–2.09) or obese (OR: 2.60, 95%CI: 1.84–3.66) subjects had a significantly higher risk of hypertension than normal weight people, and underweight was a protective factor for hypertension(OR: 0.52, 95%CI: 0.29–0.93). People with dyslipidemia have a higher risk of hypertension than those with normal lipids (OR: 3.05, 95%CI: 2.36–3.90). In addition,there was a significant potentiating interaction effect between overweight or obesity and dyslipidemia(overweight: RERI (1.91, 95%CI: 0.17–3.66), AP (0.40, 95%CI:0.14–0.66), SI (2.03, 95%CI:1.11–3.74) and obesity: RERI (2.20, 95%CI:1.01–3.40), AP (0.38, 95%CI:0.18–0.58), SI (1.84, 95%CI:1.18–2.89), while no interaction was found between underweight and dyslipidemia. Low body weight is an independent protective factor for hypertension, but overweight, obesity and dyslipidemia are risk factors for hypertension, and dyslipidemia significantly shared interactions with overweight and obesity that influenced the risk of hypertension.

## Introduction

Hypertension affects more than one billion people worldwide, and that number is increasing, which makes this disease a serious public health problem worldwide^1^. In China, the prevalence of hypertension is high and rising, but the control rate is not satisfactory. Cardiovascular disease related to hypertension is still the main cause of death in Chinese adults^2^. A study showed that 44.7% of Chinese adults aged 35–75 suffer from hypertension^3^. Therefore, identifying the risk factors for hypertension and effective prevention are essential to reduce the burden on public health.

To date, many studies have found that overweight, obesity and dyslipidemia are strongly associated with hypertension^4^. Excessive weight gain, especially weight gain associated with visceral fat gain, is the main risk factor for hypertension, accounting for 65% to 75% of the risk of essential hypertension in humans^5^. An increase in body mass index (BMI) leads to an increase in the risk of hypertension. In addition, studies have proven that a higher BMI is a risk factor for hypertension and dyslipidemia^6^. Furthermore, dyslipidemia is common in patients with hypertension, diabetes mellitus, and metabolic syndrome^7^, and elevated serum levels of total cholesterol, LDL cholesterol, and non-HDL cholesterol are all associated with an increased risk of hypertension^8^.

Hypertension is a disease with multifactorial influences, and there may be interactions among various influencing factors to exert their influence on hypertension. Previous studies have shown that the interaction between HbA1c and abdominal adiposity contributes to the development of hypertension^9^, the interaction between BMI and family history of hypertension has an impact on the risk of hypertension^10^, and there is a significant interaction between smoking and overweight with an impact on hypertension prevalence^11^. However, it is unknown whether there is an interaction between BMI and dyslipidemia to produce an effect on hypertension. Therefore, the present study used data from a cross-sectional survey to determine the associations among BMI, dyslipidemia, and hypertension and to explore the potential effect of the interaction between BMI and dyslipidemia on the prevalence of hypertension.

## Methods

### Subjects

The survey was conducted between November 2019 and January 2020 at community-based health institutions in Changsha, Hunan Province, China, with the aim of correctly assessing the major public health problems and their influencing factors in the city. The subjects were residents over 15 years old, and multistage random sampling was used to select eligible subjects. Residents under 15 years of age, pregnant women, and residents with cognitive impairment, serious illness or disability that might affect the survey were not included in the survey, and all subjects signed the informed consent form. On the day of enrollment, each subject underwent a cross-sectional survey, including a questionnaire, physical examination, and laboratory measurements. On the day before the survey, community investigators informed the subjects about their diet and other related questions and inquired the subjects on the survey day to ensure that the subjects maintained an empty stomach for at least 8 h. A total of 2740 subjects with complete data were included by multistage cluster random sampling, ranging in age from 15 to 92 years (mean age: 55.97 ± 16.40 years). Among them, a total of 765 patients with hypertension were investigated and classified as cases, and 1975 patients with normal blood pressure were classified as controls. Statistical analyses were conducted to investigate risk factors and the interactions between these factors in patients with hypertension.

### Data collection

After obtaining subjects’ written informed consent, face-to-face structured interviews were conducted by qualified interviewers to collect participants’ data. The data obtained were entered separately by two researchers and then checked against each other. The questionnaire was designed with reference to the 2017 Questionnaire Specific for Social Factors Involved in the Prevention and Control of Chronic Diseases in China, and the main contents are as follows: general information (gender, age, education level, etc.), smoking, drinking and eating conditions, physical activity, chronic disease awareness and treatment conditions. The physical examination included measurements of height, weight and waist circumference, which were measured using uniformly distributed instruments. Blood pressure was measured using a standard mercury sphygmomanometer (2 mmHg per unit), and the subjects avoided strenuous exercise, were mentally relaxed and rested quietly for 5 min before measurement. Two BP measurements were taken 1–2 min apart and averaged for records. An additional measurement was required if the first two readings differ by > 5 mmHg, and the mean value of the three readings was recorded^12^. Blood collection was conducted by the field investigation team, and all subjects fasted for at least 8 h. Hypertension was defined as follows: systolic blood pressure (SBP) ≥ 140 mmHg and/or diastolic blood pressure (DBP) ≥ 90 mmHg, or the patient was taking hypertensive medication^12^. Body mass index (BMI) was calculated as weight (kg)/height^2^ (m^2^), and subjects were classified as underweight (< 18.5 kg/m^2^), normal weight (18.5 to 23.9 kg/m^2^), overweight (24.0 to 27.9 kg/m^2^), or obese (≥ 28.0 kg/m^2^) based on Chinese criteria^13^. Dyslipidemia was defined according to the criteria of the 2007 Chinese Guidelines on Prevention and Treatment of Dyslipidemia in Adults^14^.

### Statistical methods

Statistical analysis was performed using SPSS software (version 20.0, IBM Corp, Armonk, NY, USA). Quantitative data with a normal distribution are described by their mean ± standard deviation, and the t-test was used for comparisons between groups. Categorical data are expressed as percentages (%) and were analyzed by the chi-square test (χ2 test). Unconditional logistic regression models are often used for dichotomous variables to explore the relationship between dependent and independent variables and to determine the risk or protective factors of dependent variables. In this study, a univariate logistic regression model was used to explore the significant factors affecting hypertension, and then, these important factors were applied to multivariate logistic regression analysis by the stepwise forward method. The results of multivariate logistic regression analysis and the interaction table invented by T Anderson were used to analyze the additive interaction between BMI and dyslipidemia, and the following interaction indices were calculated: (1) the relative excess risk due to interaction (RERI); (2) attributable proportion due to interaction (AP); and (3) the synergy index (SI)^15^. The 95% confidence interval (95% CI) of the interaction indicators was calculated using the table produced by T Anderson^16^. If the confidence interval of RERI or AP contains 0 or the confidence interval of SI contains 1, it indicates that the two factors have no interaction. All tests of statistical significance were two-tailed tests, and *P* < 0.05 was considered statistically significant.

### Ethics approval and consent to participate

This study was approved by the Ethics Committee of Medical College of Hunan Normal University and was in accordance with standards set forth by the Declaration of Helsinki. Written informed consent was obtained from all participants(or their parent or legal guardian in the case of children under 18).

## Results

### General Characteristics of enrolled subjects

**Figure 1:**
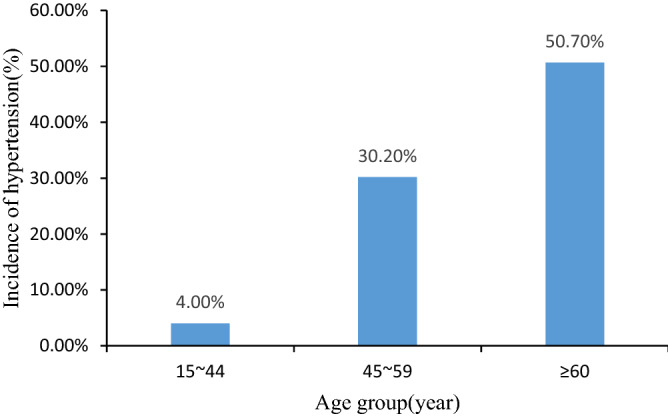
The prevalence of hypertension increases with age (*P* < 0.05).

In this cross-sectional study, 2740 participants with a mean age of 55.97 ± 16.40 years and a mean BMI of 23.48 ± 3.29 kg/m^2^ were included. A total of 765 participants (27.9%) were found to have hypertension. Table 1 shows the basic characteristics of the study participants and the prevalence of hypertension in different populations. As seen from the results in Table 1, there was a significant age difference in hypertension (*P* < 0.001), and the prevalence of hypertension increased significantly with increasing age (Fig. 1). There were also differences in the prevalence of hypertension among residents with different educational levels (*P* < 0.05), and the prevalence of hypertension decreased with increasing educational level. In addition, we found a higher prevalence of hypertension in patients with diabetes (P < 0.001) and dyslipidemia (*P* < 0.001). In terms of BMI, there were significant differences in the prevalence of hypertension among different BMI grades (*P* < 0.001), and the prevalence of hypertension increased with increasing BMI. However, no statistically significant differences were found for the following variables: gender (*P* = 0.772), residence (*P* = 0.076), smoking (*P* = 0.272), and alcohol consumption (*P* = 0.564).

**Table 1:** Analysis of basic characteristics of hypertension and nonhypertension.

Variables	Hypertension (n, %)	Nonhypertension (n, %)	t/χ2	*P* value
Age (years, mean ± sd)	66.5 ± 10.5	51.9 ± 16.5	22.726^a^	< 0.001
BMI(kg/m^2^, mean ± sd)	24.5 ± 3.4	23.1 ± 3.2	9.886^a^	< 0.001
Gender			0.084^b^	0.772
Male	318(28.2)	809(71.8)		
Female	447(27.7)	1166(72.3)		
Age			353.739^b^	< 0.001
15 ~ 44	28(4.0%)	670(96.0%)		
45 ~ 59	438(30.2%)	1014(69.8%)		
≥ 60	299(50.7%)	291(49.3%)		
Place of residence			5.158^b^	0.076
City	522(26.8%)	1424(73.2%)		
Rural	90(33.0%)	183(67.0%)		
The rural–urban junction	153(29.4%)	368(70.6%)		
Educational level			168.739^b^	< 0.001
No formal schooling	21(35.0%)	39(65.0%)		
Primary school	247(44.0%)	315(56.0%)		
Junior high school	236(30.1%)	549(69.9%)		
High school	199(27.3%)	530(72.7%)		
College degree or above	62(10.3%)	542(89.7%)		
Smoking			1.205^b^	0.272
Yes	197(29.6%)	469(70.4%)		
No	568(27.4%)	1506(72.6%)		
Drinking			0.333^b^	0.564
Yes	142(29.0%)	348(71.0%)		
No	623(27.7%)	1627(72.3%)		
Diabetes			186.344^b^	< 0.001
Yes	183(61.4%)	115(38.6%)		
No	582(23.8%)	1860(76.2%)		
Dyslipidemia			231.503^b^	< 0.001
Yes	248(58.4%)	177(41.6%)		
No	517(22.3%)	1798(77.7%)		
BMI			85.903^b^	< 0.001
Underweight	16(12.1%)	116(87.9%)		
Normal weight	341(22.9%)	1147(77.1%)		
Overweight	317(35.7%)	572(64.3%)		
Obesity	89(43.6%)	115(56.4%)		

^a^Student's t-test,

^b^Chi-square test; BMI: body mass index.

### Association between BMI or dyslipidemia and hypertension risk

Multivariate analysis showed that after adjusting for confounding factors such as sex, age, diabetes, and education, underweight was a protective factor for hypertension (OR: 0.52, 95% CI: 0.29–0.93), while overweight (OR: 1.70, 95% CI: 1.39–2.09) and obesity (OR: 2.60, 95% CI: 1.84–3.66) were risk factors for hypertension. In unifactorial logistic regression analysis, the risk of hypertension in people with abnormal blood lipids was significantly higher than that in people with normal blood lipids (OR: 4.87, 95% CI: 3.93–6.05). After adjusting for confounding factors, the risk level was reduced, but it was still a risk factor for hypertension (OR: 3.05, 95% CI: 2.36–3.90) (Table 2).

**Table 2:** Independent effects of BMI and dyslipidemia on the risk of hypertension.

Variables	Hypertension (n,%)	Nonhypertension (n,%)	X^2^	P value	OR_1_^a^ (95% CI)	OR_2_^b^ (95% CI)
BMI			85.90	< 0.001		
Normal weight	341(22.9%)	1147(77.1%)			1.00(ref)	1.00(ref)
Underweight	16(12.1%)	116(87.9%)			2.60(1.93,3.52)	0.52(0.29,0.93)
Overweight	317(35.7%)	572(64.3%)			5.61(3.11,10.14)	1.70(1.39,2.09)
Obesity	89(43.6%)	115(56.4%)			1.40(1.03,1.90)	2.60(1.84,3.66)
Dyslipidemia			231.50	< 0.001		
Yes	248(58.4%)	177(41.6%)			4.87(3.93,6.05)	3.05(2.39,3.90)
No	517(22.3%)	1798(77.7%)			1.00(ref)	1.00(ref)

^a^OR_1_ is the result of univariate analysis.

^b^OR_2_ is the result of multivariate analysis. The adjusted confounding factors included sex, age, diabetes, education level and occupation.

### Interaction between BMI and dyslipidemia

Participants were divided into four groups based on their BMI levels: underweight, normal weight, overweight or obese. Compared with normal-weight individuals without dyslipidemia, those with dyslipidemia and obesity had the highest risk of hypertension (adjusted OR: 5.82, 95% CI: 3.08–10.99), and those with dyslipidemia and overweight had a 4.77 times higher risk of hypertension than the reference group (Table 3). Furthermore, there were significant interactions between overweight or obesity and dyslipidemia (overweight: RERI (1.91, 95% CI: 0.17–3.66), AP (0.40, 95% CI: 0.14–0.66), SI (2.03, 95% CI: 1.11–3.74) and obesity: RERI (2.20, 95% CI: 1.01–3.40), AP (0.38, 95% CI: 0.18–0.58), SI (1.84, 95% CI: 1.18–2.89)). However, the adjusted OR of dyslipidemia and low body weight was not statistically significant (2.37, 95% CI: 0.66–8.56), indicating that they had no statistical significance on the risk of hypertension, and the interaction indicators showed no additive interaction between the two (Table 4).

**Table 3:** The interaction between BMI and dyslipidemia on the risk of hypertension.

Variables		Hypertension (n,%)	Nonhypertension (n,%)	OR_1_^a^ (95% CI)	OR_2_^b^ (95% CI)
Dyslipidemia	BMI				
No	Normal weight	253(19.3)	1061(80.7)	1.00(ref)	1.00(ref)
	Underweight	10(8.3)	111(91.7)	0.38(0.20,0.73)	0.43(0.22,0.87)
	Overweight	200(28.4)	503(71.6)	1.67(1.35,2.07)	1.49(1.18,1.88)
	Obesity	52(34.7)	98(65.3)	2.23(1.56,3.24)	2.26(1.50,3.39)
Yes	Normal weight	88(50.6)	86(49.4)	4.30(3.10,5.96)	2.36(1.64,3.39)
	Underweight	6(54.5)	5(45.5)	5.04(1.53,16.64)	2.37(0.66,8.56)*
	Overweight	117(62.9)	69(37.1)	7.12(5.13,9.88)	4.77(3.32,6.85)
	Obesity	37(68.5)	17(31.5)	9.17(5.06,16.49)	5.82(3.08,10.99)

^a^OR_1_ is the result of univariate analysis.

^b^OR_2_ is the result of multivariate analysis. The adjusted confounding factors included sex, age, diabetes, education level and occupation.

*Not statistically significant.

**Table 4:** Indicators of interaction between BMI and dyslipidemia.

Variables		RERI(95%CI)	AP(95%CI)	SI(95%CI)
Dyslipidemia	Underweight	− 1.30(-4.80, 2.20)	− 0.55(− 2.66, 1.56)	0.51(0.05, 5.13)
Dyslipidemia	Overweight	1.91(0.17, 3.66)	0.40(0.14, 0.66)	2.03(1.11, 3.74)
Dyslipidemia	Obesity	2.20(1.01, 3.40)	0.38(0.18, 0.58)	1.84(1.18, 2.89)

RERI: the relative excess risk due to interaction; AP: attributable proportion due to interaction; SI: the synergy index.

## Discussion

Body mass index (BMI) is an important indicator of body fat and can correct for the effect of height on body mass. Studies have shown that BMI is the most sensitive physical measure to predict high blood pressure compared with other common indicators of obesity^17^. Many studies have confirmed that in Chinese adults, overweight and obesity are important risk factors for hypertension^18,19^, and overweight/obesity can cause metabolic imbalance and hormone level changes in the human body, resulting in high blood pressure^20^. Additionally, dyslipidemia is closely related to hypertension, and dyslipidemia can change the permeability of cell membranes by affecting their structure and can also cause renal microvascular damage, which can lead to hypertension^21^. Studies have shown that elevated levels of serum levels of total cholesterol (TC), low-density lipoprotein cholesterol (LDLC) and high-density lipoprotein cholesterol (HDLC) are associated with an increased risk of hypertension^8^.

The results of this study found that people with diabetes or dyslipidemia are at higher risk for hypertension and that both overweight and obesity are risk factors for hypertension. This is similar to the findings of Zhang FL’s study, which found that individuals who were overweight/obese and had dyslipidemia or diabetes had a higher risk of developing hypertension^22^. Hashemi Madani N found that hypertension, dyslipidemia, and impaired fasting blood glucose were all likely to lead to an increase in adverse cardiovascular events^23^. de Lombera Romero F found that hypertension was often associated with other atherosclerosis risk factors, such as dyslipidemia, insulin resistance and obesity^24^. Moreover, Hu L found that in South China, the prevalence of hypertension increased in overweight and obese people^25^. This is because compared with people with normal BMI, overweight and obese people are more likely to have metabolic syndrome, such as fat metabolism disorder and insulin antibody, which leads to the occurrence of cardiovascular diseases such as hypertension. Moreover, sustained and moderate weight loss may help lower blood pressure in the long term^26^.

In addition, the present study verified the additive interaction between dyslipidemia and overweight or obesity. Jian S’s studies showed that the triglyceride index and obesity had an interactive effect on the incidence of hypertension in middle-aged and elderly people, and dyslipidemia and obesity were both risk factors for hypertension^27^. Related studies have shown that dyslipidemia and overweight/obesity have several common mechanisms for increasing blood pressure; for example, dyslipidemia can impair arterial endothelial function, leading to hypertension ^28^. Previous studies have proven that obesity and the cardiovascular disease family have an interactive effect on hypertension^29^. There is an interaction between dyslipidemia and oral contraceptives that can increase the risk of high blood pressure^30^. However, few studies have explored whether there is an interaction between dyslipidemia and overweight/obesity in hypertension. Therefore, based on the findings in this study that overweight/obesity and dyslipidemia interact with hypertension, further studies on the interaction mechanism between these factors are needed in the future.

There are still some limitations in this study. For example, information on bad behaviors such as smoking and drinking was obtained by asking the respondents, which may have information bias. In addition, the data analysis was based on cross-sectional studies, which could not distinguish time sequences and limited causal inference.

## Conclusion

In conclusion, underweight is an independent protective factor for hypertension, but overweight, obesity and dyslipidemia are risk factors for hypertension. Moreover, dyslipidemia significantly shared interactions with overweight and obesity that influenced the risk of hypertension. Therefore, people who are overweight or obese and suffer from dyslipidemia are at higher risk of hypertension. It is necessary to take concrete measures in the early prevention and control of hypertension. Moreover, lifestyle intervention and health guidance should be carried out as soon as possible to reduce the incidence of hypertension in high-risk groups.

## Data Availability

The datasets used and/or analyzed during the current study are available from the corresponding author on reasonable request.

## Abbreviations

BMI: Body mass index
RERI: Relative excess risk due to interaction
AP: Attributable proportion due to interaction
SI: Synergy index
OR: Odds ratio
CI: Confidence interval
